# Decoding Carbapenem Resistance: Detection of Carbapenemase Genes in Clinical Isolates of Carbapenem-Resistant Acinetobacter baumannii

**DOI:** 10.7759/cureus.103938

**Published:** 2026-02-19

**Authors:** Neha Mamgain, Barnali Kakati, Vijay Kumar, Nupur Koul, Akhilesh Kumar

**Affiliations:** 1 Microbiology, Himalayan School of Biosciences, Swami Rama Himalayan University, Dehradun, IND; 2 Microbiology, All India Institute of Medical Sciences, Rishikesh, Rishikesh, IND; 3 Biotechnology, Himalayan School of Biosciences, Swami Rama Himalayan University, Dehradun, IND; 4 Microbiology, Himalayan Institute of Medical Sciences, Swami Rama Himalayan University, Dehradun, IND

**Keywords:** blandm-1, blaoxa-51, carbapenem resistance, carbapenem-resistant acinetobacter baumannii, combined disc test, crab, metallo-beta-lactamase

## Abstract

Introduction

*Acinetobacter baumannii* is a common nosocomial pathogen that has developed multidrug resistance (MDR) to different classes of antibiotics, including carbapenems. The World Health Organization has declared carbapenem-resistant *A. baumannii *(CRAB) a critical priority pathogen.

Aims and objective

This study aimed to determine the antimicrobial susceptibility of CRAB, identify carbapenemase production, and detect carbapenemase genes in clinical isolates of CRAB.

Methods

This study was conducted in the Department of Microbiology, Himalayan Institute of Medical Sciences and School of Biosciences, Swami Rama Himalayan University, Dehradun. Antimicrobial susceptibility and identification were performed by the VITEK-2 automated system (bioMérieux, Marcy-l'Étoile, France). Carbapenemase production was determined by using the combined disc test (CDT) method. These isolates were genetically screened for carbapenemase genes.

Results

A total of 100 CRAB isolates were included in the study. All 100 (100%) isolates were resistant to β-lactam/β-lactamase inhibitor combinations, cephalosporins, fluoroquinolones, and aminoglycosides. The highest sensitivity was observed for minocycline (15/100, 15%), followed by cotrimoxazole. Phenotypic detection of carbapenemase production was carried out using the CDT, followed by molecular confirmation through polymerase chain reaction (PCR). Carbapenemase production was observed in 97 (97%) of CRAB isolates. *bla*_OXA-51_, *bla*_NDM-1_, *bla*_OXA-23_, and *bla*_VIM_ were detected in 100 (100%), 94 (94%), 88 (88%), and 70 (70%) of isolates, respectively. Coexistence of *bla*_NDM-1 _and *bla*_OXA-23_ (83, 83%) as well as *bla*_NDM-1_ and *bla*_VIM _(65, 65%) among CRAB isolates was a notable finding in our study. The relationship between the presence of carbapenemase genes and antibiotic susceptibility test results was evaluated using the chi-square test, with p-values <0.05 considered statistically significant.

Conclusion

In our study, CRAB isolates demonstrated high resistance to antimicrobial agents, with limited sensitivity to minocycline and cotrimoxazole. The coexistence of multiple carbapenemase genes, including *bla*_NDM-1_, *bla*_OXA-23_, and *bla*_VIM_, reflects significant genetic diversity and enhances the potential for horizontal gene transfer and rapid dissemination within healthcare settings. Such high-level gene coexistence has important clinical and epidemiological implications, as it may contribute to treatment failure and hospital outbreaks. This finding emphasizes the critical need for strict infection control measures, antimicrobial stewardship programs, and continuous molecular surveillance of resistance determinants to limit the spread of these MDR organisms.

## Introduction

The genus *Acinetobacter* includes strict Gram-negative, aerobic, non-fermenting, catalase-positive, and oxidase-negative coccobacilli [1]. It comprises more than 50 species. The majority of them are non-pathogenic, while some are pathogenic. *Acinetobacter baumannii* is the most common pathogenic species. Other species with pathogenic potential include *A. calcoaceticus*, *A. lwoffii*, *A. haemolyticus*, and *A. johnsonii*. Among these, *A. baumannii* emerged as an opportunistic pathogen with increased morbidity and mortality [2].

Increased prevalence of multidrug-resistant (MDR) *A. baumannii *strains globally has complicated the therapeutic management of these pathogens [3]. Moreover, emerging carbapenem resistance has led to limited selection of carbapenems as therapeutic agents against carbapenem-resistant *Acinetobacter baumannii* (CRAB) [4].

Production of carbapenemases belonging to Ambler class A, class B, and class D is the main contributor to resistance to carbapenem drugs [5]. Non-carbapenemase-mediated resistance involves loss of outer membrane porins and upregulation of efflux pumps to reduce drug accumulation [6]. A high resistance pattern among CRAB is attributed to genomic plasticity, which permits the acquisition of novel resistance genes via mobile genetic elements and the overexpression of antimicrobial resistance genes (ARGs) [4].

CRAB is mainly mediated by plasmid-encoded antibiotic resistance genes [7,8]. Class A (e.g., *bla*_KPC_) and class B metallo-β-lactamases (*bla*_NDM_, *bla*_IMP_, *bla*_VIM_ ) are commonly plasmid-borne and transmissible, whereas class D OXA-type enzymes include intrinsic *bla*_OXA-51_ and acquired variants such as *bla*_OXA-23_, *bla*_OXA-24/40_, and *bla*_OXA-58_ [9,10].

Although phenotypic assays are widely used for carbapenemase detection, they may lack sufficient sensitivity and specificity to accurately identify resistance genes in CRAB isolates. Moreover, regional molecular data in our setting are limited, highlighting the need for a comprehensive evaluation of resistance mechanisms.

Therefore, the primary objective of this study was to investigate the mechanisms of carbapenem resistance among clinical isolates of CRAB in a tertiary care hospital in the sub-Himalayan region. Specifically, we aimed to detect carbapenemase production and identify major carbapenemase-encoding genes using phenotypic and molecular methods.

## Materials and methods

Study design

This prospective observational study was conducted in the Department of Microbiology, Himalayan Institute of Medical Sciences and School of Biosciences, Swami Rama Himalayan University, from May 2022 to April 2024 after obtaining the institutional ethical clearance. A total of 100 non-repetitive CRAB isolates were included in the study.

Inclusion criteria

Clinical isolates of* A. baumannii* received in the microbiology laboratory that exhibited carbapenem resistance, as determined by antibiotic susceptibility testing interpreted according to the Clinical and Laboratory Standards Institute (CLSI) guidelines (CLSI M100-ED32/2022), were included in the study.

Exclusion criteria

Carbapenem-sensitive isolates, duplicate isolates, and mixed cultures were excluded from the study.

Ethical approval

The study protocol was approved by the Ethics Committee of the Himalayan Institute of Medical Sciences, Swami Rama Himalayan University, Dehradun, under approval number SRHU/HIMS/ETHICS/2022/391, dated November 22, 2022.

Isolation

The clinical samples were subjected to culture on blood and MacConkey agar for overnight incubation at 37°C. Colony morphology was assessed on plating media, and Gram-negative coccobacilli were identified by Gram staining. All culture media and consumables were purchased from HiMedia Laboratories, Mumbai, India.

Identification and antimicrobial susceptibility testing by the Vitek-2 System

Identification and determination of antimicrobial susceptibility testing (AST) of the clinical isolates were performed using the VITEK-2 system (bioMérieux, Marcy-l'Étoile, France) (GNID card, AST-406 card), following CLSIM100-ED32/2022 guidelines [11]. Wherever required, the Kirby-Bauer disc diffusion method was used for selected antimicrobials. On the basis of colony morphology and Gram staining, appropriate VITEK identification and AST cards were used. A total of 100 non-repetitive CRAB clinical isolates were selected based on confirmed carbapenem resistance. American Type Culture Collection (ATCC) 19606 *A. baumannii* was used as the control strain.

Phenotypic detection of carbapenemases

Combined Disc Test (CDT)

**Figure 1:**
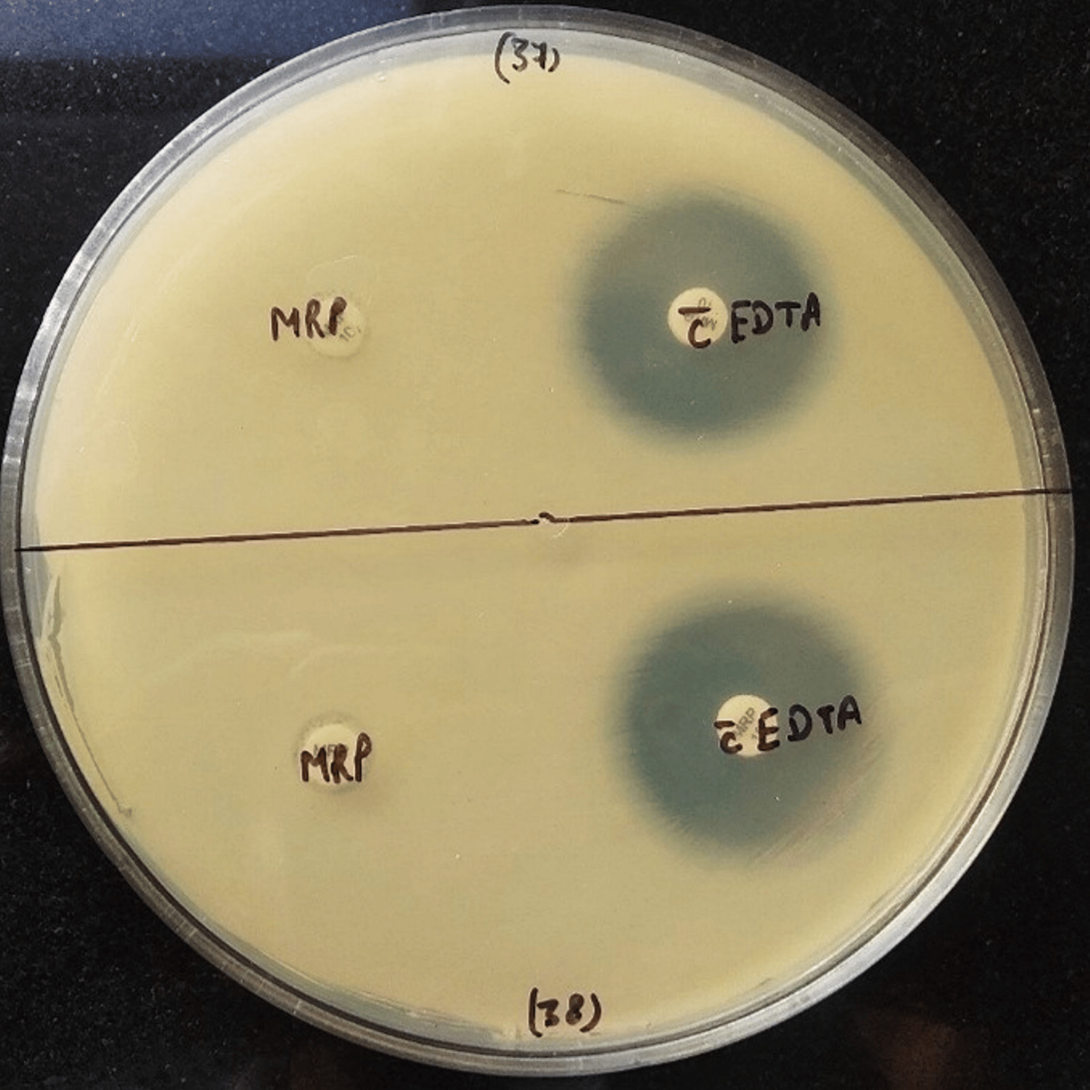
Combined disc test (CDT). Zone size >7 mm around the EDTA-impregnated meropenem disc indicates a CDT-positive test. EDTA: ethylenediaminetetraacetic acid.

The CDT was conducted in accordance with a comparable study previously described [12]. A lawn culture of test isolate (0.5 McFarland opacity standard) was done on Mueller-Hinton agar. Two 10 µg meropenem discs were placed on inoculated plates. A total of 10 µL of 0.5 M ethylenediaminetetraacetic acid (EDTA) solution was added to one of the meropenem discs. After overnight incubation at 35 ± 2°C for 16-18 hours, if the zone of inhibition of EDTA-impregnated meropenem discs compared to meropenem alone is >7 mm, the test was considered positive. CDT test-positive isolates were identified as carbapenemase-producing (metallo-beta-lactamase) *A. baumannii* strains (Figure 1).

Genotypic characterization

Polymerase chain reaction (PCR) assays for the detection of carbapenemase genes were performed using a set of primers previously described in the literature (Table 1) [2,5].

**Table 1:** Primer sequence for genes. *bla*: β-lactamase gene; *bla*_KPC_: Klebsiella pneumoniae carbapenemase; *bla*_NDM-1_: New Delhi metallo-β-lactamase-1; *bla*_VIM_: Verona Integron-encoded metallo-β-lactamase; *bla*_IMP_: Imipenemase (metallo-β-lactamase); *bla*_OXA-51_: Oxacillinase-51; *bla*_OXA-23_: Oxacillinase-23; *bla*_OXA-48_: Oxacillinase-48; F: forward primer, R: reverse primer; bp: base pairs.

Genes	Sequence	Amplicon size
*bla*_KPC_	F: 5’-CGTCTAGTTCTGCTGTCTTG -3’ R: 5’-CTTGTCATCCTTGTTAGGCG-3’	798 bp
*bla*_NDM-1_	F: 5’-GGTTTGGCGATCTGGTTTTC-3’ R: 5’-CGGAATGGCTCATCACGATC-3’	621 bp
*bla*_VIM_	F: 5’-GATGGTGTTTGGTCGCATA-3’ R: 5’- CGAATGCGCAGCACCAG-3	390 bp
*bla*_IMP_	F: 5’-GGAATAGAGTGGCTTAAYTCT-3’ R: 5’-CGGTTTAAYAAAACAACCACC-3’	232 bp
*bla*_OXA-51_	F: 5’-AGTGAAGCGTGTTGGTTAT-3’ R: 5’-CAGCCTACTTGTGGGTYTA-3	285 bp
*bla*_OXA-23_	F: 5’-TTTACTTGCTATGTGGTTGCT-3’ R: 5’-ATCACCTGATTATGTCCTTGA-3’	107 bp
*bla*_OXA-48_	F: 5’-GCGTGGTTAAGGATGAACAC-3’ R: 5’-CATCAAGTTCAACCCAACCG-3’	438 bp

Deoxyribonucleic acid (DNA) extraction was done by the bacterial DNA extraction Kit (HiMedia, Mumbai, India) under the manufacturer’s protocol. PCR amplification was carried out in a 20 µL reaction mixture containing 2 µL template DNA, 0.4 µL Taq DNA polymerase, 1 µL dNTP mix, 2 µL 1X Taq buffer (1.5 mM MgCl₂), and 1 µL of each primer (0.5 µM final concentration). Initial denaturation was done at 94°C for three minutes for each gene, followed by 35 cycles of denaturation, annealing, and extension. Previously confirmed carbapenemase-producing isolates have been used as controls. Positive and negative controls were included in all PCR assays. PCR product bands were analyzed after electrophoresis in a 1.5% agarose gel at 75 V for 45 minutes in 1X Tris-acetate-EDTA (TAE)-containing ethidium bromide (EtBr) under ultraviolet (UV) radiation (Table 2).

**Table 2:** PCR conditions for carbapenemase genes. *bla*: β-lactamase gene; *bla*_NDM-1_: New Delhi metallo-β-lactamase-1; *bla*_IMP_: Imipenemase metallo-β-lactamase; *bla*_VIM_: Verona Integron-encoded metallo-β-lactamase; *bla*_OXA-51_: Oxacillinase-51; *bla*_OXA-23_: Oxacillinase-23; *bla*_OXA-48_: Oxacillinase-48; *bla*_KPC_: Klebsiella pneumoniae carbapenemase.

Genes	Denaturation	Annealing	Extension	Final extension
*bla*_NDM-1_	94°C x 30 s	66.7°C x 1 min	72°C x 45 s	72°C x 10 min
*bla*_IMP_	94°C x 1 min	65°C x 1 min	72°C x 2 min	72°C x 1 min
*bla*_VIM_	94°C x 30 s	65°C x 30 s	72°C x 1 min	72°C x 5 min
*bla*_OXA-51_	94°C x 1 min	56°C x 1 min	72°C x 2 min	72°C x 1 min
*bla*_OXA-23_	94°C x 1 min	57°C x 1 min	72°C x 2 min	72°C x 1 min
*bla*_OXA-48_	94°C x 1 min	70.5°C x 1 min	72°C x 2 min	72°C x 1 min
*bla*_KPC_	94°C x 1 min	71°C x 1 min	72°C x 2 min	72°C x 1 min

Data analysis

Data analyses were performed using IBM SPSS version 20 (IBM Corp., Armonk, NY). The chi-square test was used to determine the relationship between antimicrobial resistance (AMR) and ARGs.

## Results

The majority of CRAB isolates were recovered from respiratory secretions (63, 63%), followed by blood (21, 21%), pleural fluid (5, 5%), swab (2, 2%), pus (2, 2%), umbilical venous catheter tip (UVC) (2, 2%), and urine (2, 2%). Specimens contributing 1% each included tissue, ascitic fluid, and central venous catheter tip (CVC). Table 3 highlights the antimicrobial susceptibility of CRAB isolates. Out of 100 CRAB test isolates, a high level of resistance was observed against β-lactam/β-lactamase inhibitors (100, 100%), third- and fourth-generation cephalosporins (100, 100%), fluoroquinolones (100, 100%), and aminoglycosides (100, 100%). However, limited sensitivity was observed for minocycline (15, 15%), followed by cotrimoxazole (5, 5%) (Table 3).

**Table 3:** Antimicrobial susceptibility pattern of carbapenem-resistant A. baumannii (n = 100).

Antibiotic	Resistant	Intermediate	Sensitive
Ampicillin-sulbactam	99 (99%)	-	1 (1%)
Ceftazidime	100 (100%)	-	-
Cefepime	100 (100%)	-	-
Ciprofloxacin	100 (100%)	-	-
Levofloxacin	100 (100%)	-	-
Gentamicin	99 (99%)	-	1 (1%)
Amikacin	100 (100%)	-	-
Piperacillin tazobactam	100 (100%)	-	-
Cotrimoxazole	95 (95%)	-	5 (5%)
Minocycline	65 (65%)	20 (20%)	15 (15%)
Colistin	1 (1%)	99 (99%)	-
Cefoperazone - sulbactam	99 (99%)	1 (1%)	-

The prevalence of metallo-beta-lactamase (MBL) production by CDT among CRAB was as follows: 97 (97%) identified as MBL positive and three (3%) as MBL negative (Figure 1).

Out of 100 CRAB clinical isolates, the most predominant carbapenemase genes identified were *bla*_NDM-1_ (94, 94%) and *bla*_OXA-23_ (88, 88%), apart from the intrinsic* bla*_OXA-51_ (100, 100%) (Table 4).

**Table 4:** Distribution of the carbapenemase genes in carbapenem-resistant A. baumannii isolates (n = 100). *bla*_OXA-23_, *bla*_OXA-48_, and *bla*_OXA-51_: Oxacillinase genes; *bla*_NDM-1_: New Delhi metallo-β-lactamase-1; *bla*_VIM_: Verona integron-encoded metallo-β-lactamase; *bla*_IMP_: Imipenemase metallo-β-lactamase; *bla*_KPC_: Klebsiella pneumoniae carbapenemase.

Genes	Frequency, n(%)
*bla*_OXA-51_	100 (100%)
*bla*_OXA-23_	88 (88%)
*bla*_OXA-48_	0 (0%)
*bla*_NDM-1_	94 (94%)
*bla*_VIM_	70 (70%)
*bla*_IMP_	0 (0%)
bla_KPC_	0 (0%)
Two gene combinations	
*bla*_NDM-1_, *bla*_VIM_	65 (65%)
*bla*_NDM-1_, *bla*_OXA-23_	83 (83%)
*bla*_VIM_,* bla*_OXA-23_	63 (63%)
Three or more gene combinations	
*bla*_NDM-1_, *bla*_VIM_, *bla*_OXA-23_	59 (59%)

The majority of isolates were obtained from intensive care unit (ICU) samples (71, 71%), among which 93 (93%) harbored *bla*_NDM-1_, followed by 87 (87%) carrying *bla*_OXA-23_.

Association of *bla*_NDM-1_ and *bla*_OXA-23_ genes with antimicrobial susceptibility profile showed no statistically significant correlation with resistance to the tested antibiotics. Most isolates remained resistant irrespective of the presence or absence of these carbapenemase genes (Table 5).

**Table 5:** Association of carbapenemase genes with antimicrobial susceptibility profile. A/S: ampicillin-sulbactam; PIT: piperacillin-tazobactam; GEN: gentamicin; COT: cotrimoxazole; Mi: minocycline; CST: colistin; NA: not applicable; *bla*_OXA-23_: oxacillinase; *bla*_NDM-1_: New Delhi metallo-β-lactamase. A p-value <0.05 is considered statistically significant. The chi-square test was applied to calculate the p-value.

Drugs	AST	*bla*_NDM-1_		P-value	*bla*_OXA-23_		P-value
		Present	Absent		Present	Absent	
A/S	S	1	0	0.940	1	0	0.880
	R	93	6		87	12	
PIT	S	0	0	NA	0	0	NA
	R	94	6		88	12	
GEN	S	1	0	0.940	1	0	0.880
	R	93	6		87	12	
COT	S	5	0	0.729	5	0	0.520
	R	89	6		83	12	
Mi	S	15	0	0.298	14	1	0.817
	I	20	0		17	3	
	R	58	6		57	8	
CST	I	93	6	0.940	88	11	0.120
	R	1	0		0	1	

## Discussion

The global emergence of MDR *A. baumannii*, particularly CRAB, poses a major therapeutic challenge in clinical practice and underscores the importance of regional surveillance. In the present study, CRAB isolates were predominantly recovered from respiratory tract samples. Comparable findings were reported by Guddeti et al., who observed 50% of CRAB isolates from endotracheal secretions, while Norris et al. reported 44.8% of *A. baumannii* isolates from urine [13,14]. Variations in isolation rates may be attributed to factors such as the organism’s natural colonization of the respiratory tract and its role as a major causative agent of respiratory infections, making it one of the most commonly recovered pathogens from respiratory specimens [13].

In this study, CRAB isolates showed uniform resistance to aminoglycosides, fluoroquinolones, cephalosporins, and β-lactam/β-lactamase inhibitor combinations. These findings align with previous reports showing high variability in resistance rates, ranging from 8.8% to 92.1%, depending on the antibiotic class and geographical region, likely reflecting differences in antimicrobial usage patterns [15-17].

Minocycline showed the highest sensitivity, followed by cotrimoxazole, indicating limited therapeutic options. The high prevalence of MBL-producing CRAB isolates, detected by the CDT, is consistent with other studies reporting 82-97% positivity [18,19]. Among MBL genes,* bla*_NDM-1_ was the most common, followed by *bla*_VIM_, while *bla*_IMP_ was absent. These genes are often carried on mobile genetic elements, facilitating horizontal spread [20,21].

In addition to *bla*_OXA-51_, the intrinsic resistance gene *bla*_OXA-23_ was the most prevalent class D carbapenemase gene found in CRAB clinical isolates. This enzyme efficiently hydrolyzes carbapenems and other β-lactams, contributing significantly to resistance [22,23]. Although some studies have reported 100% prevalence of *bla*_OXA-23_, our findings suggest ongoing dissemination, which may be due to various plasmids and genetic structures [24,25].

Our data highlight the dominance of class B and D β-lactamases in CRAB, with a high co-occurrence of *bla*_NDM-1_, *bla*_VIM_, and *bla*_OXA-23_, underscoring the complexity of resistance mechanisms. These findings support previous studies and reinforce the need for robust surveillance and routine MBL screening in diagnostic laboratories [26,27]. The high frequency and coexistence of carbapenemase genes observed in this study may be attributed to plasmid-mediated horizontal gene transfer and sustained antibiotic selection pressure in critical care settings. Such gene clustering enhances dissemination potential and limits therapeutic options. The presence of multiple β-lactamases in a single isolate complicates both diagnosis and treatment, especially in settings with limited access to last-line drugs like colistin and polymyxin. Regular use of phenotypic confirmatory methods, as used here, offers a simple and cost-effective strategy for early detection.

In our study, antibiotic resistance was not significantly associated with the presence of carbapenemase genes, as determined by the chi-square test, suggesting the possible involvement of alternative non-carbapenemase-mediated resistance mechanisms. Similarly, Anggraini et al. also reported an insignificant relationship between the presence of antibiotic resistance genes and phenotypic resistance among clinical isolates of *A. baumannii *[26].

The high resistance of CRAB isolates to major antibiotic classes, including limited sensitivity to minocycline and cotrimoxazole, highlights a serious therapeutic challenge, especially in ICU settings. Early detection through routine phenotypic and molecular methods, as done in this study, is crucial for timely treatment. Although this study provides valuable insights, its single-center nature calls for broader, multicentric research to inform regional and national strategies.

This study has certain limitations that should be considered when interpreting the findings. As a single-center study, the findings may not be generalizable to other settings with different antimicrobial usage patterns. The limited sample size may have affected the detection of less prevalent resistance genes. The absence of molecular typing restricted the assessment of clonal relatedness and transmission dynamics. Additionally, only selected resistance genes were evaluated, and other mechanisms may have contributed to the observed resistance patterns. Lastly, the study did not evaluate patient outcomes in relation to resistance profiles, which could have added clinical context to the findings. Therefore, further large-scale multicenter studies with comprehensive genomic approaches are needed to validate and expand upon these findings.

## Conclusions

This study reveals a high burden of MBL-producing CRAB isolates in our tertiary care setting, underscoring the growing public health threat of multidrug-resistant *A. baumannii*. The frequent coexistence of key carbapenemase genes highlights the complexity of resistance and the potential for rapid dissemination in healthcare environments. These findings emphasize the need for strengthened antimicrobial stewardship, strict infection control measures, and ongoing regional surveillance. Future studies should focus on genomic characterization and multicenter surveillance to better understand resistance evolution and transmission dynamics.

## References

[1] Zalts R, Neuberger A, Hussein K, Raz-Pasteur A, Geffen Y, Mashiach T, Finkelstein R (2016). Treatment of carbapenem-resistant Acinetobacter baumannii ventilator-associated pneumonia: retrospective comparison between intravenous colistin and intravenous ampicillin-sulbactam. Am J Ther.

[2] Eldegla HE, Nour I, Nasef N (2021). Molecular characterization of carbapenem resistant Gram-negative rods in neonatal intensive care unit of Mansoura University Children’s Hospital. Afr J Microbiol Res.

[3] (2025). CDC. 2019 antibiotic resistance threats report. http://www.cdc.gov/DrugResistance/Biggest-Threats.html.

[4] Elbrolosy AM, Labeeb AZ, Hassan DM (2019). New Delhi metallo-β-lactamase-producing Acinetobacter isolates among late-onset VAP patients: multidrug-resistant pathogen and poor outcome. Infect Drug Resist.

[5] Yang Q, Rui Y (2016). Two multiplex real-time PCR assays to detect and differentiate Acinetobacter baumannii and non- baumannii Acinetobacter spp. carrying blaNDM, blaOXA-23-like, blaOXA-40-like, blaOXA-51-like, and blaOXA-58-like genes. PLoS One.

[6] Amala Reena AA, Subramaniyan A, Kanungo R (2017). Biofilm formation as a virulence factor of Acinetobacter baumannii: an emerging pathogen in critical care units. J Curr Res Sci Med.

[7] Hasanin A, Mukhtar A, El-Adawy A, Elazizi H, Lotfy A, Nassar H, Ghaith D (2016). Ventilator associated pneumonia caused by extensive-drug resistant Acinetobacter species: colistin is the remaining choice. Egypt J Anaesth.

[8] Kyriakidis I, Vasileiou E, Pana ZD, Tragiannidis A (2021). Acinetobacter baumannii antibiotic resistance mechanisms. Pathogens.

[9] Takebayashi Y, Findlay J, Heesom KJ, Warburton PJ, Avison MB, Evans BA (2021). Variability in carbapenemase activity of intrinsic OxaAb (OXA-51-like) β-lactamase enzymes in Acinetobacter baumannii. J Antimicrob Chemother.

[10] Manchanda V, Sanchaita S, Singh N (2010). Multidrug resistant acinetobacter. J Glob Infect Dis.

[11] Clinical and Laboratory Standards Institute (2022). CLSI M100. Performance Standards for Antimicrobial Susceptibility Testing. https://clsi.org/shop/standards/m100/.

[12] Pattnaik A, Banashankari GS (2017). Assessment of biofilm production in carbapenem resistant Acinetobacter species isolated from different clinical specimens. J Med Sci Clin Res.

[13] Guddeti PK, Shah H, Karicheri R, Singh L (2023). Clinical profile of patients and antibiogram of Acinetobacter baumannii isolates in a tertiary care hospital, central India. J Pure Appl Microbiol.

[14] Norris SC, Pandya HB, Pipaliya BP, Javadekar TB (2024). Antimicrobial resistance patterns in Acinetobacter baumannii: a study from a tertiary care center in Vadodara, Gujarat. Indian J Microbiol Res.

[15] Reddy D, Morrow BM, Argent AC (2015). Acinetobacter baumannii infections in a South African paediatric intensive care unit. J Trop Pediatr.

[16] Agyepong N, Govinden U, Owusu-Ofori A, Essack SY (2018). Multidrug-resistant gram-negative bacterial infections in a teaching hospital in Ghana. Antimicrob Resist Infect Control.

[17] Kempf M, Rolain JM (2012). Emergence of resistance to carbapenems in Acinetobacter baumannii in Europe: clinical impact and therapeutic options. Int J Antimicrob Agents.

[18] Bonnin RA, Rotimi VO, Al Hubail M, Gasiorowski E, Al Sweih N, Nordmann P, Poirel L (2013). Wide dissemination of GES-type carbapenemases in Acinetobacter baumannii isolates in Kuwait. Antimicrob Agents Chemother.

[19] Falcone M, Tiseo G, Leonildi A (2022). Cefiderocol- compared to colistin-based regimens for the treatment of severe infections caused by carbapenem-resistant Acinetobacter baumannii. Antimicrob Agents Chemother.

[20] Karthikeyan K, Thirunarayan MA, Krishnan P (2010). Coexistence of blaOXA-23 with blaNDM-1 and armA in clinical isolates of Acinetobacter baumannii from India. J Antimicrob Chemother.

[21] Ramirez MS, Bonomo RA, Tolmasky ME (2020). Carbapenemases: transforming Acinetobacter baumannii into a yet more dangerous menace. Biomolecules.

[22] El-Badawy MF, Abdelwahab SF, Alghamdi SA, Shohayeb MM (2019). Characterization of phenotypic and genotypic traits of carbapenem-resistant Acinetobacter baumannii clinical isolates recovered from a tertiary care hospital in Taif, Saudi Arabia. Infect Drug Resist.

[23] Ren G, Zhou M, Ding N, Zhou N, Li Q (2016). Analysis on distribution features and drug resistance of clinically isolated Acinetobacter baumannii. Exp Ther Med.

[24] Benamrouche N, Lafer O, Benmahdi L (2020). Phenotypic and genotypic characterization of multidrug-resistant Acinetobacter baumannii isolated in Algerian hospitals. J Infect Dev Ctries.

[25] Kazi M, Nikam C, Shetty A, Rodrigues C (2015). Dual-tubed multiplex-PCR for molecular characterization of carbapenemases isolated among Acinetobacter spp. and Pseudomonas spp. J Appl Microbiol.

[26] Anggraini D, Santosaningsih D, Saharman YR (2022). Distribution of carbapenemase genes among carbapenem-non-susceptible Acinetobacter baumanii blood isolates in Indonesia: a multicenter study. Antibiotics (Basel).

[27] Alkasaby NM, El Sayed Zaki M (2017). Molecular study of Acinetobacter baumannii isolates for metallo-β-lactamases and extended-spectrum-β-lactamases genes in Intensive Care Unit, Mansoura University Hospital, Egypt. Int J Microbiol.

